# Badminton instructional in Malaysian schools: a comparative analysis of TGfU and SDT pedagogical models

**DOI:** 10.1186/s40064-016-2872-3

**Published:** 2016-07-29

**Authors:** Sanmuga Nathan

**Affiliations:** Faculty of Sports and Coaching, Sultan Idris Education University, Tanjung Malim, Malaysia

**Keywords:** Teaching Games for Understanding, Movement to base, Skill execution, Decision-making, Teachers’ reflection

## Abstract

Model based physical education curriculum of Teaching Games for Understanding (TGfU) is still at early stage of implementation in Malaysian schools whereby the technical or skill-led model continues to dominate the physical education curriculum. Implementing TGfU seems to be problematic and untested in this environment. Therefore, this study examined, the effects that a revised model of TGfU compared to Skill Drill Technical (SDT) a technical model had on learning movement skills in Badminton, including returning to base, decision making and skill execution whilst performing in a doubles game play and also explored teachers’ perceptions of navigating between the two models. Participants aged 15.5 ± 1.0 years, *N* = 32, school Badminton players were randomly selected and assigned equally into groups of TGfU and SDT. Reflective data was gathered from two experienced physical education teachers who were involved in this study. Findings indicated for movement to the base in doubles game play indicated significant improvement, after intervention via TGfU. As for decision-making and skill execution in doubles game play, analysis revealed no significant difference after intervention. Findings from teachers reflection, indicated the importance of mini game play in both TGfU and SDT models, as the students enjoyed, and built up positive attitudes for both winning or losing in game situations. However, when negotiating the TGfU model, the teacher found it difficult at times to execute the pedagogical model, as students needed guidance to discuss aspects related to tactics. However, to keep this pedagogical model viable further research findings ought to be circulated among teachers in Malaysia and similar Southeast Asian counties.

## Background

Badminton is the national sport in Malaysia and an important game in that country’s physical education curriculum; however, it is still being taught using a skill-based approach based on the secondary schools (KPM 2002). In contrast, many places around the world including, Europe, Canada, Australia and other seemingly more research-aware countries, have moved on from a skill-led approach to a tactics led approach, in both the teaching and coaching contexts. There has also been a shift in the research paradigm amongst authors with the majority of research into skills-based learning becoming largely irrelevant (Metzler 2005). However, in the Malaysian context making comparisons between the tactical approach and technical approach is still a lively issue for debate, as the teachers who are accustomed to the “old fashioned” technical-skill based ways-of-doing are now starting to be challenged by innovation (Nathan and Haynes 2013)

In countries such as Malaysia, in order to disseminate information about a tactical pedagogical approach, such as the revised TGfU model in a physical education badminton game context, it is essential that some form of research needed to be undertaken especially to answer top ten research questions related to TGfU (Memert et al. 2015). Consequently, this research is being addressed, having as its basis previous instructional research for the game of badminton with the provision of some modifications, using the revised TGfU model linking with CLA. The research project included the examination of important badminton game play parameters such as movement to the base (a position on the court in badminton that is regarded as a “ready” position), decision making, and skill execution in game play performances (1996b).

An additional point to consider was the emotional aspects associated with game play. Much of the literature and research undertakings in badminton are based around physiological and bio-mechanical information (Phomsoupha and Laffaye 2015), and limited investigations have been carried out to examine evidence of the emotions, particularly as observed through player expressions such as smiling (happiness) and other non-verbal expressions, such as those representing disappointment. Crocker et al. (2002) highlighted the importance for players to express a sense of celebration, after an important occasion, such as scoring, with smiles, hugs, and other “happy” expressions. In contrast, the emotional experience of the losing team is often clearly expressed in their facial expressions

It should be noted that the original Teaching Games for Understanding (TGfU) and the revised model, are both underpinned by the constructivist perspective of learning. In contrast, more recently motor learning researchers have linked TGfU with CLA that is underpinned by ecological teaching and learning models (Clemente et al. 2012; Renshaw et al. 2010). The benefit of this revised approach forges a partnership with the constraints-led focus that can provide students with new opportunities and new sources of learning and motivation (Clemente et al. 2012). Even though the pedagogical principle of representation and exaggeration in TGfU do appear to have some form of task and constraints, the research findings are inconclusive.

Apart from the work of French et al. (1996b), whose research contained many important and crucial badminton parameters of game play, limited research has been conducted in terms of badminton game play, practitioner or teacher’s beliefs and perceptions concerning non-linear pedagogy, including for example an approach that partners TGfU with a constraints led approach, compared to a linear pedagogical skill led approach. Stolz and Pill (2014) pointed out this schism when they reported that many different opinions existed amongst teachers-as-practitioners when compared to the claims made by academics. Even though the TGfU model has become a prominent and prevalent feature within research, its impact within the practices of teaching games in physical education class has yet to be fully explored, especially in relation to implementing tactical game teaching, which seems to be a very challenging proposition (Pearson et al. 2005; Rink et al. 1996). Consequently according to Pearson et al. (2005) linear pedagogy, or skill-led, or the technical models of teaching still dominate teaching games in physical education class.

The revised TGfU and the original version of TGfU seem to promote tactical intelligence and players’ levels of creativity using questioning strategies. Empirical research of game intelligence is being developed via convergent tactical thinking skills, and tactical creativity through divergent tactical thinking skills, especially during the early stages of youth sports development in team sports like soccer, basketball, hockey, and handball (Memmert et al. 2010; Wein 2004). With this notion in mind perhaps badminton playing countries in Asia, including for example Malaysia and Indonesia that favour the skill-led or the linear pedagogy, need to re examine their pedagogical methodology in order to avoid taking a backseat in future international rankings as no badminton players from Malaysia and Indonesia holds top five ranking in the world except Lee Chong Wei (Badminton World Federation 2016).

An appropriate pedagogical model plays an important role in the teaching and learning of improving game play components and producing game-smart players (Light 2013). Playing badminton, hockey, soccer and basketball, for a example requires players to have a good command, and a high order of game knowledge that permits quick decision-making as to “what to do” and “how to do” tactics, agility, speed and accuracy in executing skills at the appropriate time in a game play situation (Gréhaigne et al. 2001; Light 2003; Siedentop 2001). In reference to badminton components such as footwork, movement to base, skill execution (contact, drop shot, smash, clear and drive), it is cognitive-thinking-tactical decision making that is crucial in game play performance (Moody et al. 2014).

**Fig. 1:**
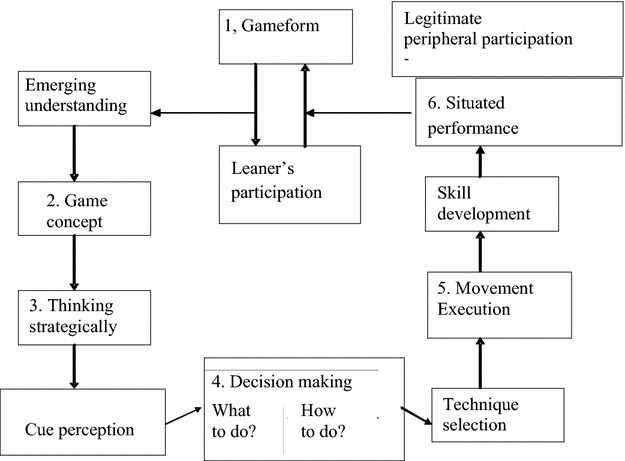
Revised TGfU model (Kirk and Macphail 2002)

The original model of TGfU coined by Bunker and Thorpe (1982) was introduced as an alternative to more traditional skill-led approaches, which is sometimes referred to as the technical model approach (Kirk and Macphail 2002). The original TGfU model underpins information processing and constructivist theories, which revolve around six teaching steps, these are: (i) understanding game form; (ii) game appreciation; (iii) tactical awareness; (iv) making appropriate decisions, about what tactics to use and how to utilize tactics- appropriate skills in small sided game play by the players; (v) how to execute skills that are technically sound; and finally (vi) upgrading game performance by employing all these steps. However, the original TGfU model still lacked elements of educational learning theory. Kirk and Macphail (2002) detected that the six steps of learning from original TGfU model still had important parameters missing such as thinking strategically, cue perception and technique selection, which are crucial in a student’s learning process. As a result a revised model of TGfU was suggested. This TGfU revised model included the missing parameters such as perception cues, so that players could think strategically pertaining to when and how to use specific tactics and strategies as reflected in Fig. 1.

**Fig. 2:**
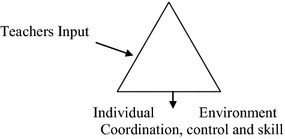
Constraints—led theory (Newell 1986)

Motor learning theory advocates the importance of a constraints-led perspective for acquisition of movement skills and game play knowledge. The motor learning proponents argue that the constraints–led framework can assist physical educators to built their teaching and learning instruction using task, performer and environmental constraints to explain how learners acquire movement skills and decision making behaviors. The constraints-led approach was based on the discipline of ecological psychology and Bernstein’s dynamical system’s theory (Renshaw et al. 2010). The constraints–led theory as shown in Fig. 2, is divided into three categories, namely, the performer, environment and task, as it is the interaction of these factors that shape students’ behaviors. Of note it was the work of Newell (1986), which provided a framework for understanding how movement patterns emerge during a task performance.

As shown in Fig. 2, the performer represents the functional characteristics of learners and factors related to their physical, physiological, cognitive and emotional status, whereby the learner’s morphology, fitness level, technical abilities and psychological factors, such as anxiety level and motivation, may shape the way individuals’ approach a movement task (Renshaw et al. 2010; Newell 1986).

Blomqvist et al. (2000) examined the effect the original TGfU had on game understanding and game performance between young expert and novice players in badminton. Findings indicated the young expert players were significantly better than the novice players in terms of long serve and clear, performing longer shots, backhand shots, and physically they travelled significantly longer distances around the court, and they also demonstrated better understanding of tactics. Again Blomqvist et al. (2001) investigated the effects of two forms of instruction traditional with TGfU strategy versus traditional approach on students’ knowledge, game understanding, skill execution and game performance. Analysis of results showed that for badminton knowledge, game understanding, and skill and game performance that the strategy (TGfU) orientated and traditional group improved their badminton knowledge, whereas the control group showed no improvement. Furthermore the traditional group significantly improved in the skill of serving, however, for forceful shots (hit into target area) and cooperative shots (hit straight to the opponents-no tactical) there was no significant mean effect.

As far as badminton is concerned, Jianyu and Wenhao (2012, p. 29) studied badminton skills and tactics across four development skill levels in game play performance. The analysis of the results indicated that, when skill level improved, a large portion of low quality serves and strokes across all the skill levels were low, and in the rates of using standard serves and strokes, forceful strokes, and return to home base were also low. These findings signal to teachers the importance of learning all the components of badminton, and linking players’ developmental age to skill levels, but more importantly how to blend these two facets via appropriate pedagogical approaches.

On the other hand, Long and Lung (2014) employed action research to explore the original TGfU, with regard to their cognitive performance and learning within a game situation. The findings indicated that students understood TGfU instructions, and their knowledge of badminton and learning also demonstrated progress. An analysis of the findings also revealed there was a positive response to increased knowledge of the game, more enjoyment in playing sports and improved exercise habits. However, teachers did have some dilemmas, in trying to grasp the concepts of the TGfU model, such as teaching time, control of students, designating teaching activities for students, problems with information and providing instruction, and their students’ attitudes and knowledge affecting their teaching processes.

Sheppard (2014) investigated TGfU using three types of instructions: (i) the hybrid model (combination of original TGfU with Hellison’s Levels of Personal and Social Responsibility, PSR), (ii) TGfU, (Bunker and Thorpe 1986) and (iii).Mr. A’ own way type of TGfU intervention. She found that all three instructions were meaningful in developing personal and social responsibly behaviors, perceived responsibility, all about badminton game play irresponsibility in action, a positive learning environment, and a learned response. Noting that this research was undertaken based on the original model rather than the TGfU revised model. O’Leary (2015) investigated how experienced teachers delivered TGfU and those factors that influenced informal learning of this instructional model. Findings revealed the traditional or linear pedagogy approach to teaching games learned from childhood and partially learned in higher education were ‘washed out’ by the influence of teaching a student-centered approach to teaching games. This study indicated TGfU seems to be have greater potential compared with the traditional approach provided that circumstances conducive to learning and there was sufficient time.

A syntheses of literature revealed that to date limited research has been conducted into the effect of the combination or partnership between the TGfU revised model with CLE, and its impact compared to more a traditional linear pedagogy, namely, skills, drill, and tactics (SDT) in badminton game play.

There is no exception to these findings in Malaysia, as anecdotal evidence from the Malaysia Physical Education Curriculum for secondary schools still appears to purport a skill-based (SDT) the linear approach even though there were some revisions made in the curriculum in primary school curriculum reference to TGfU (KPM 2002, 2010; Nathan and Ratnavdivel 2012).

More research-based evidence is required to answer the question whether teachers and curriculum planners understand how to implement the game play approach via TGfU revised model with CLA as a non-linear pedagogy. As it stands in Malaysia, most badminton lessons are conducted using a linear pedagogical approach comprising the familiar; warming up, followed by skills teaching, mini game play and finally with limbering down, coupled with an authoritarian teaching style exhibited by the teacher. These observations are in line with the linear model of instruction, noted by Metzler (2005) and Rink (2002) that does not comply with the non-linear approach based on Metzler’s benchmark. Again from anecdotal observation it appears that many students learning badminton in Malaysia in the physical education setting seem to find it difficult make appropriate decisions using specific tactics and skills in doubles game situations.

Few investigations have been conducted by Malaysian teachers, researchers or coaches into the effectiveness of the TGfU orginal model examples hockey (Nathan 2015; Nathan and Haynes 2013) and Handball (Balakrishnan et al. 2011a). However, almost no research has been conducted in Malaysia using TGfU revised model by Kirk and MacPhail (2002) to evaluate the effectiveness as student-centered teaching on students learning badminton in terms of game play via tactics, strategies or skills. Besides that, limited research has been undertaken into teachers’ perceptions of their own experiences in teaching students during badminton competitions, or the student’s emotional and cognitive level during “on the spot” teacher questioning, or the even the teacher’s questioning approach.

The intended purpose of this study was to: (i) investigate the effectiveness the TGfU revised with CLA as a non-linear model compared to the linear model of Skill Drill Tactical (SDT) in badminton. Specifically, movement skill to base, tactical decision-making (contact, drop shot, smash, clear and drive) and skill execution (contact, drop shot, smash, clear and drive) in doubles badminton game play among Malaysian school following 12 units of instruction. (ii) Examine via semi structured reflective journal entries the reflections and experiences of teachers utilizing the TGfU revised model with CLA on game play, cognitive and emotional learning and the challenge of effective teacher’s questioning to stimulate learning.

The following hypotheses and research question pertain to teaching the sport of badminton using two different pedagogical approaches: The non-linear approach, namely, the TGfU revised model partnership CLA compared to the linear approach, i.e., the skill-led approach known as SDT in this research.

1. To what extent do the players’ in TGfU with CLA and SDT model differ in terms of movement skills in returning to base in doubles game play before and after interventions?
2. To what extent are players in either the TGfU with CLA or SDT model different in terms of decision making regarding shot selection (contact, drop shot, smash, clear and drive) in doubles game play performance before and after interventions?
3. In terms of skill execution (contact, drop shot, smash, clear and drive) in doubles game play performance are there any differences before and after intervention in players in either the TGfU with CLA or SDT groups?
4. What were the comments and experiences of the two (*n* = 2) teachers who implemented the different pedagogical models in terms of their students’ game play, cognitive development and emotional learning? What were the challenges involved with creating effective questions in order to stimulate student learning?

## Methods

### Participants

The sample consisted of thirty-two school students aged 15.5 ± 1.0 years with equal numbers of males (*n* = 16) and females (*n* = 16), who were assigned equally to two groups one to TGfU, with equal numbers of males and females, and the second group to the SDT model, with the same gender allocations. The players have no experience playing badminton using the TGfU approach. Informed consent was obtained from all students (*n* = 32) and their parents or guardians through their teachers. This intervention was investigated from two different settings, specifically in the States of Perak and Penang in Malaysia. Two qualified physical education teachers with more than 10 years of involvement in badminton were selected to teach the two groups using the two contrasting pedagogical models, prior to intervention both teachers were given modules and briefing on these two pedagogical models.

The main methodology was an experimental balanced group design using pre and posttests. This study also investigated the teachers’ reflections and experiences, when their students were competing in doubles game play. The semi structured reflection framework was derived from work of Jarrett (2011). The two teachers were requested to record reflections about their perceptions and experience utilizing TGfU and SDT. A structured journal was provided for this purpose. The journal’s reflective framework included space for comments about game play, cognitive and emotional learning and the challenge of effective teacher questioning using divergent questioning (when, how, why, what, who and when) to build higher order thinking and stimulate learning undertaken. This approach was adapted from the work undertaken by Jarrett (2011), whereby data were gleaned from reflective journals kept by the teachers.

The study was carried out over for a period of 5 weeks for intervention protocols during 12 badminton lessons. An additional period of 2 weeks was needed to collect and analyze pretest and post-test data.

**Fig. 3:**
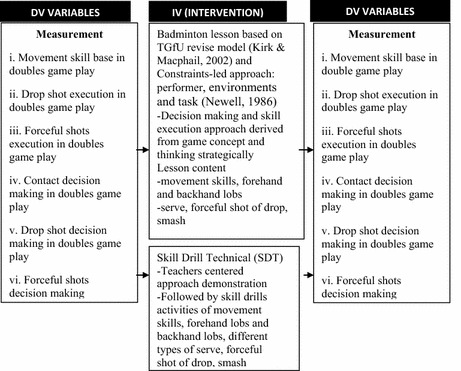
Conceptual framework TGfU revised model and SDT model

The conceptual framework shown in Fig. 3 illustrates the interventions, partnership model viz TGfU with CLA compared SDT model as an independent variables and game play parameters as dependent variables.

### Testing procedures

As for game play procedures, prior to playing the doubles games the students were familiarized with the court markings and boundaries. Pairs of students from TGfU revised model and SDT groups were assigned to play pairs of their opponents. There were 8 pairs in TGfU revised model and another 8 pairs SDT. They played 8 doubles games at pretest and again at post-test. Games were recorded by two automatic video cameras, as permission amd ethical consideration granted by Education Ministry of Malaysia. The doubles game was played for 10 min, with the time controlled by a research assistant.

This study adapted and adopted the game play observational instrument used by ten other researchers (French et al. 1996a; Mitchell et al. 2005; Turner and Martinek 1999) to examine skill components and cognitive-decision making of game performance. The dependent variables of base movement skill, skill execution of contact, drop shot, forceful shot (clear, drive and smash) and decision making of contact, drop shot, forceful shots (clear, drive and smash) were coded 5, 4, 3, 2 or 1. Where 5 = very effective performance; 4 = effective performance, usually; 3 = moderately effective performance, sometimes; 2 = very weak performance and 1 = very weak performance, or never. The following Table 1 shows a summary of the variables.

**Table 1:** Summary of the calculations for the dependent variables calculated as measures of game play

Measures	Description	Code range
Movement skill base	Total number of appropriate movements to base between skill execution	Coded 5, 4, 3, 2 for success or 1 for weak/never
Contact execution	Total number of clean contacts with the shuttle	Coded 5, 4, 3, 2 for success or 1 for weak/never
Drop shot execution	Total number of drop shots including overhead clear using slicing, hitting, pushing techniques, with the shuttle	Coded 5, 4, 3, 2 for success or 1 for weak/never
Forceful shots	Total number of forceful shots (clear, drive, and smash)	Coded 5, 4, 3, 2 for success or 1 for weak/never
Contact decision	Total number of appropriate contact decisions	Coded 5, 4, 3, 2 for success or 1 for weak/never
Drop shot decision	Total number of appropriate drop shot decisions	Coded 5, 4, 3, 2 for success or 1 for weak/never
Forceful decision	Total number of appropriate clear, drive and smash decisions	Coded 5, 4, 3, 2 for success or 1 for weak/never

The two experienced and qualified Malaysian badminton teachers were trained to code all the dependent variables using the game play observational instrument whilst watching both the videotaped doubles game play situations. Regarding inter coder reliability, based on the 8 players featured in two doubles game situations, the agreement between the coder and principal researcher was 82 % for movement skill base, 78 % for skill execution (contact, drop shot, forceful shot) and 84 % for decision making of contact, drop shot, and forceful shot.

### Qualitative data generation

The two teachers involved in the study were requested to record their reflections for three sessions of their teaching using TGfU revised model and SDT model. For each session the teachers recorded their reflections in terms of how involved the student’s became in game play, cognitive and emotional learning and the challenge of effective teachers questioning (Jarrett 2011; Pearson and Webb 2008).

### The TGfU revised model group

The experimental intervention comprising badminton lessons were: tactics (opening and closing the space, defending as a pair, wining the point); skills; various movement skills or footwork; forehand and backhand lobs; as well as serve, appropriate forcefulness of the drop shot and, smash skills. The TGfU revised model with the constraint-led approach was used to impart these tactics and skills as part of the content of the badminton lessons. Based on TGfU revised model (Kirk and Macphail 2002) lessons highlighted ‘what to do’, ‘how to do, and ‘when to do’, and linked game concepts with strategic thinking, cue perceptions skill selection.

The badminton lessons were formulated to take into consideration the situational and background understanding of badminton game play. The lessons were carried out in the following sequence over period of 5 weeks. Firstly, with a brief discussion about a selected topic linking the game concept and strategic thinking, involving tactics and skills (3–5 min). Then a warming up session (7 min), next, a game situation (10 min) whereby the performer or the players are given a simple game task or problem to solve, and then a short briefing followed by brief recovery of 3 min. Then a second game situation was undertaken, during which more complex and difficult constraints tasks or game problems are allocated for the players to solve (10 min), followed by a finally limbering down and feedback activity (5 min). The TGfU revised model with the constraints-led approach lessons were carried out using a tactical approach focused on the badminton skills of serve, contact, drive, clear, drop, smash and movement of returning to base based on tactical complexity level 1, 2 and 3 (Mitchell et al. 2013) via guided problem solving method.

### The SDT linear pedagogy group

The linear pedagogy of SDT model (Technical model) was based on the common practice in Malaysia of predominantly using a combination of skill drills activities and towards end of the lesson utilizing a tactical approach mini game situation. Skill and drill activities were based on the model proposed by Rink (2002) as this conceptual framework emphasizes the importance of teaching and learning skills prior to game play through skill drills practice (French et al. French et al. 1996a, b). Lesson topics were based on the skills of serve, contact, drive, clear, drop, smash and movement of returning to the base using direct approach to instruction.

### Treatment validity

The following steps were undertaken to maintain the fidelity for the implementation of the two teaching models. Initially, the principal researcher conducted a simultaneous briefing session for the teachers about how to implement the two different models. Then the two teachers were provided with teaching modules of TGfU with CLA and SDT, plus a checklist, which contained a summary of the two pedagogical models. Next, the principal researcher demonstrated the implementation of these teaching interventions and the method of carrying out all the required test measures. Finally, the principal researcher, to ensure the teachers conducted the teaching units accordingly, provided a preliminary briefing session. The students underwent 2 lessons per week comprising 40 min per lesson for 5 weeks as the teaching intervention. One group, designated, Group A underwent the non-linear TGfU revised model with CLA approach to learning, while the second, Group B, were engaged in linear pedagogy using a teacher centered teaching, namely, the SDT model.

The implementation of revised TGfU model with CLA was undertaken based on the Metzler (2005) benchmark. Metzler (2005), cited by Parry (2014) proposed that teachers who engaged with game based approaches (GBAs), such as TGfU, needed to fulfill eight tactical games benchmarks, namely: creating a tactical problem as the organizing center for learning tasks; the teacher needs to begin the unit segment with a game form to assess student knowledge; the teacher needs to identify tactical and skill areas from game form; the teacher has to use deductive questions to get students to solve a tactical problem; the teacher uses clear communication for situated learning tasks; the teacher uses high rates of guidance and feedback during situated learning tasks, assessment; a tactical problem for students to solve is introduced; and, small sided/modified games are used within lessons that sufficiently reflect a pedagogy that emphasize each of the core principles of Game Centered Approaches (GCAs). Whereas the implementation of SDT was based on the technical model framework set by Rink (2002).

### Data analysis

In order to measure the effectiveness of TGfU and SDT model performance outcomes, the dependent variables of movement skill to base, decision making (contact, drop shot, smash, clear and drive), skill execution (contact, drop shot, smash, clear and drive) in doubles game play were analyzed by ANOVA using SPSS version 2.1 software. In addition, ANCOVA (using pre-test score as covariate) was used to confirm the results when there was a significant difference at base line level.

The generated qualitative data were analyzed based on an inductive methodology. The data gathered from the reflective journals were organized and coded via inductive coding procedures suggested by Thomas (2006) and the framework suggested by Jarrett (2011). The inductive procedures were chosen to allow significant themes to emerge from the two teachers who taught TGfU revised model and SDT. Data were generated in terms of game play, cognitive and emotional learning and the challenge of effective teacher questioning to stimulate learning undertaken; adapted from the work of Jarrett (2011). The labeled categories of themes such game play, cognitive and emotional learning and the challenge of effective teacher questioning were predetermined to reduce overlap and redundancy and maintained the themes in lime suggestion by Creswell (2013)and Lincoln and Guba (1985).

## Results and discussions

The results for the quantitative analysis are presented within three sub headings based upon each of the quantitative research questions. These are:

### Movement to *base* in doubles game play (2 vs. 2)

**Fig. 4:**
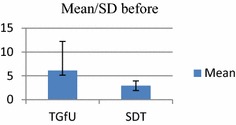
Pretest for movement to base in doubles

**Fig. 5:**
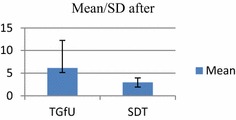
Post-test for movement to base in doubles

Analysis of results indicated for the pretest, among school badminton players, there was no significant difference between TGfU and SDT models on movement to base in 2 versus 2 doubles game play, F(1, 30) = 1.94, p > 0.05. *TGfU*, (M/SD: 2.10 ± 1.10 and SDT, M/SD: 4.00 ± 5.45). For the post-test occasion, however, analysis indicated a statistically significant difference between the two groups. An inspection of the following scores showed that the TGfU outperformed the SDT. TGfU (M/SD: 6.13 ± 6.10) and SDT model (M/SD: 2.93 ± 1.00), F(1, 30) = 4.30, p < 0.05. Table 2 and Figs. 4 and 5 show the results of movement to base in 2 versus 2 doubles game play.

**Table 2:** Pre-test and post-test for movement to base in double game play

Model	*Mean*	*SD*	*N*	*p*
Pretest				
TGfU	2.10	1.10	16	*F*(1, 30) = 1.94, *p* > 0.05
SDT	4.00	5.45	16	
Posttest				
TGfU	6.13	6.10	16	*F*(1, 30) = 4.30, *p* < 0.05
SDT	2.93	1.00	16

### Skill execution in doubles game play

**Fig. 6:**
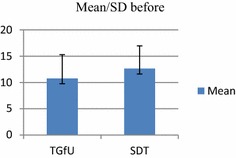
Pretest skill execution in doubles

**Fig. 7:**
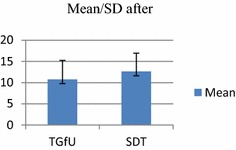
Posttest skill execution in doubles

Analysis of results indicated no significant difference between TGfU with SDT models on skill execution in doubles 2 versus 2 game play at pre-test among school badminton players, *F*(1, 30) = 0.346, *p* > 0.05, TGfU, (*M/SD*: 11.56 ± 4.41 and SDT, *M/SD*: 10.87 ± 1.54). Post-test analysis also indicated no significant difference between TGfU (M/SD: 16.75 ± 5.89) and SDT models (*M/SD*: 14.75 ± 5.50), *F*(1, 30) = 0.984, *p* > 0.05. Table 3 and Figs. 6 and 7 show the results for skill execution outcome for doubles game play.

**Table 3:** Pre-test and post-test for skill execution in double game play

Model	*Mean*	*SD*	*N*	*P*
Pretest				
TGfU	11.56	4.41	16	*F*(1, 30) = .346, *p* > 0.05
SDT	10.87	1.54		
Posttest				
TGfU	16.75	5.89	16	*F*(1, 30) = .984, *p* > 0.05
SDT	14.75	5.50	16

### Decision making in doubles game play

**Fig. 8:**
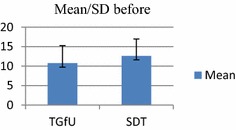
Pretest decision making in doubles

**Fig. 9:**
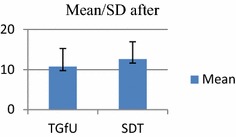
Posttest decision making in double

Findings revealed no significant difference between TGfU and SDT models on decision making in 2 versus 2 game play at pre-test among school badminton players, *F*(1, 30) = 0.031, *p* > 0.05, TGfU, (*M/SD*: 9.25 ± 4.02 and SDT, *M/SD*: 9.50 ± 4.00). Post-test result also indicated no significant difference between TGfU (M/SD: 12.75 ± 4.52) and SDT models (*M/SD*: 12.62 ± 4.34), *F*(1, 30) = 0.006, *p* > 0.05. Table 4 and Figs. 8, 9, show the results for decision-making outcome in 2 versus 2 doubles game play.

**Table 4:** Pre-test and post-test for decision making in double game play

Model	*Mean*	*SD*	*N*	*P*
Pretest				
TGfU	9.25	4.02	16	*F*(1, 30) = 0.31, *p* > 0.05
SDT	9.50	4.00		
Posttest				
TGfU	12.75	4.52	16	*F*(1, 30) = .006, *p* > 0.05
SDT	12.62	4.34	16

### Qualitative data results

This research study utilized two teachers, identified as Teacher A, who was involved in TGfU revised model and Teacher B, assigned to the SDT intervention. Following is a brief summary of their journal entries pertaining to game play, cognitive development, emotions, and questioning strategy.

In terms of game play, Teacher A reiterated, that at times it was difficult for students to discuss the tactical elements of badminton game play, without teacher guidance and the offering of clues, as the following quote indicates:

But at times it was difficult to make the student understand, especially discussing tactics, unless I guided them with clues. (ii) Subsequently they were required to apply some of the tactics discussed in game play, for about five minutes. As they were playing, I moved from one court to another court to discuss the cues about, how to execute skills such as clear, drop shot and smash.

Conversely, Teacher B expressed the opinion that the students were simply waiting to enjoy the game play and the end of the lesson, as they spent most of their learning time practicing badminton skill drills, which the teacher contended that at times made them feel lethargic and bored with continually doing skill drills, as quote below:

I must admit that, there were times the players enjoy the badminton skill drills, but at certain of the lesson they experienced fatigue, slowed down the tempo of executing the skill drills including the serve, smash or clearance.

With regard to cognitive development, Teacher A concluded that only after many episodes of situational game play practice, were students able to improve their tactical game thinking. Whereas Teacher B claimed teaching SDT style enhanced cognitive development during skill drill session as student selected to ask questions during feedback and they were able grasp some knowledge through discussion.

As far as the emotional dimension was concerned, throughout TGfU, Teacher A claimed that intervention via situational game play, showed both winning and losing teams were equally able to exhibit a positive and enjoyable outlooks. Similarly, Teacher B reported that the students seemed to be excited when they were allowed to play mini games that occurred towards at the end of the lesson.

Based on the reports by Teacher A, concerning questioning strategy about tactics and skill, most of the “who, what, when, where, how” (WH) type questions about tactics and skills that they were able to be answered by the students as they related to their experience to playing and watching badminton at home and in the community. Notwithstanding, Teacher B reported that questions related to skill execution in badminton, the students were able to answer when requested by the teacher. Table 5 of “Appendix” provides a brief excerpt from the journals of the two teachers.

To reiterate the purpose of this study was to evaluate whether differences in three skill components of badminton were evident when a TGfU revised model with a constraints-led approach was adopted compared to the well-known traditional teaching linear model known as the SDT model in this research.

This discussion is presented in sections based on the research hypotheses and research questions. As far as movement to the base in 2 versus 2 doubles game play, analysis of results indicated significant improvement was shown by the TGfU revised model (6.13 ± 6.10) intervention compared to linear model of SDT (2.93 ± 1.00), *F*(1, 30) = 4.30, *p* < 0.05. Perhaps the achievement due to the TGfU revised model partnership with CLA to certain extent. As the CLA is characterized more holistically in three categories of constraints, namely, task, environment and performer compared to pedagogical principle of representation and exaggeration in TGfU. Task in this study refers to activities, lesson plans, organization of activities, problem solving and so on that are prepared for the players. Environment refers the ‘outside’ factors such interaction between players in the same team, anticipation of opposing players, the situational context where learning and playing, competition taking place, or the setting. The third category refers to the performer in terms of an individual’s physical-abilities, psychological readiness and social-emotional make up. Therefore, the given tasks and constraints of badminton lessons should linked with an authentic badminton playing situation in order for the students to improve the badminton outcome performance. The tasks or game lessons arranged in TGfU through small sided or mini game activities. The small sided game play environment underpins with discussion problem solving activities, the lesson or tasks consists of different degree constraint. These include challenge the performer or players using half court or full court, modified equipment, reflective discussion, guided discovery, questioning and answering via Wh questions based on game play context. However, the players able to relate their background experience while performing badminton skills using TGfU approach. Many local communities have a strong traditional connection with the game, and influence the environmental constraints by favoring the player from such an environment. As a consequence using TGfU meant the task, and environment helped the players in TGfU group improved their movement skills, specifically when moving the base.

However, the findings for movement to base contrasts with the earlier research of Blomqvist et al. (2000) with regard to game play performance in terms of dependent variables pertaining to percentages of a successful shot (hit within the boundaries of play), forceful shots (hit into target area) and cooperative shot (hit straight to the opponents-non tactical). These authors found no significant mean effect and interaction among groups on game play variables.

As for post-test results for skill execution (contact, drop shot, smash, clear and drive), analysis indicated no significant difference between TGfU (16.75 ± 5.89) and SDT model (14.75 ± 5.50), *F*(1, 30) = 0.984, *p* > 0.05.). Post-test results for decision making (contact, drop shot, smash, clear and drive), also indicated no significant difference between TGfU (12.75 ± 4.52) and SDT model (12.62 ± 4.34), *F*(1, 30) = 0.006, *p* > 0.05. The findings of skill execution and decision making in contrast with findings of movement skill to base improved significantly. This change was probably due to the movement skills to base was a comparatively simpler skill compared to complex skills of execution and tactical decision making of contact, drop shot, smash, clear and drive. Therefore these parameters of skill execution and decision making might need a longer period, i.e. more than 5 weeks of interventions for the partnership model (TGfU revised and constraints-led approach) to show its real potential.

The outcome of this research in terms of badminton skill execution of contact, drop shot, smash, clear and drive as well as decision making with regard to contact type: drop shot; smash; clear, and drive, produced findings similar to those of French et al. (1996b). Namely, that a 3 weeks intervention resulted in no significant difference between the skill group and strategy tactical group in terms of forceful shots, game decisions (other than serve), contact decisions, serve decisions, and cooperative shots as well of skill execution. However, this presents findings a contrast with the results of studies of Handball (Balakrishnan et al. 2011b), Badminton French et al. (1996b) of a six-week badminton program and found that the skill-only group improved in game performance decisions that were similar to the tactical group. Notwithstanding, French et al. (1996b) found forceful shots strongly correlated with appropriate game decision making. The present findings of the study presented in this paper indicated that for both skill execution of contact, drop shot, smash, clear and drive and decision making of contact, drop shot, smash, clear and drive there was no significant improvement after intervention using the TGfU revised approach, which could also be attributable to the initial low skill level of the participants and shorter period of intervention.

These current findings are in line with those of Jianyu and Wenhao (2012) whereby the investigation indicated that apart from the type of learning model, skill levels are an important element in game play performance. For this present study, the findings related to skill execution and decision making in 2 versus 2 doubles game play, are in line earlier findings of Blomqvist et al. (2001). These authors found that the traditional group was able to significantly improve the skill of the badminton serve. On the other hand as game play performance in terms of dependent variables’ percentages of a successful shot (hit within the boundaries of play), forceful shots (hit into target area) and cooperative shot (hit straight to the opponents-non tactical) there was no significant mean effect and interaction among groups on game play variables.

Findings elicited from a qualitative approach gleaned through data from the two teacher’s reflective journal entries in term of game play showed, interestingly enough, they had both highlighted the actual game play scenario. With regard to the TGfU revised model with partnership constraints-led approach, one teacher wrote “at times it is difficult for students to discuss the tactical game plays, unless with teacher guidance”. This statement is in line with the findings of Díaz-Cueto et al. (2010) indicating physical education teachers have difficulties when attempting to implement TGfU in terms of planning, feelings of insecurity and lack of confidence in their pedagogical approach. Furthermore, their finding revealed that after the first stage teachers’ feelings of stratification improved as the low skill students made significant improvement in decision-making and game and tactical problem solving.

With guidance the students in the non-linear pedagogy groups were able to answer WH type questions and could relate their experience to playing badminton at in their local home environment. In addition they were able to solve given badminton problems compared to those students in linear learning groups. These finding supports the non-linear TGfU revised model in partnership with a constraints-led approach, as this non-linear pedagogy emphasized the influence of situational environmental support in the learning process. This finding is in line and corroborates with that of O’Leary (2015), Chatzipanteli et al. (2015), Kirk and MacPhail (2002) and Clemente et al. (2012).

The findings of Long and Lung (2014) are matched in this present research, which showed that teachers encountered some constraints when trying to understand and implement a TGfU revised model with a constraints-led approach. They could foresee some dilemmas such as teaching time and class control, designating teaching activities for students, problems with information and giving instruction to students, and student’s attitudes and knowledge. The findings related to this present study regarding teacher dilemmas, applied not only to those who utilized the TGfU revised model, but also to those who employed the partnership with constraints-led approach.

Conversely, the teacher negotiating the linear pedagogy SDT intervention, namely, Teacher B clearly stipulated the importance of game play as reflected by comments from students waiting for some form of competitive game. It is noteworthy that this scenario seems to be universal, as game play appears to be an integral part of any physical education lesson. Support for this is found in the results of Long and Lung (2014) work who revealed there was a positive attitude to increased student’s knowledge of game play, enjoyment of sports and cultural exercise habits.

This study’s findings also revealed that both the teachers agreed to the importance of game play situations to enhance student motivation, enjoyment, and to promote positive emotions as well as making their behavior easier to manage. Teacher A considered that the TGfU revised model and partnership constrains-led game play activities to be invaluable for students in terms of building up human values within the affective learning domain. To quote Teacher A, with regard to non-linear pedagogy TGfU revised model and constraints-led approach “the winning team and losing team were equally able to perform with an enjoyable outlook”. Similarly, Teacher B reported that the students in the linear pedagogy of SDT group “seem to be excited when they were allowed to play a mini game towards at the end of the lesson.” A similar finding was reported by Robinson (2011), Lauder (2001), Chai (2009) and Nathan and Haynes (2013) who reported that enjoyment in games was associated with game competence, not just with fun via a play practice approach such TGfU. Heywood (2001) described enjoyment as an emotional state that serves as powerful source of internal motivation, and Plitz (2006) also commented that the affective domain plays an important role in games education. Interestingly the findings contrast with those reported by Snoxell (2014) who claimed that by using self-teaching and command style teaching of badminton for year 7 boys (13 years of age) that little enjoyment was exhibited.

## Conclusion

As for the primary purpose, the findings of this study generally support the previous research conducted in badminton by French et al.(1996a) and Blomqvist et al. (2001) regarding the importance of badminton game play parameters and the game based pedagogical approach such as TGfU.

Furthermore, this study highlights some insights into answering the inconclusive question of whether the TGfU revised model and partnership constraints–led approach preposition the disparity between researcher and teacher as noted by Stolz and Pill (2014). Based on the notes elicited from two teacher’s reflective journals, especially those of Teacher A who was using the TGfU revised model and partnership with constraints-led approach, which indicated that when students were asked questions in regard to tactics and skills they were able to relate answers from their experience and involvement in badminton at home and the local community. This experience extended further to even watching one of their badminton heroes on television. This finding is line with the notion pointed out by Gil et al. (2014) that the teaching–learning process in sport has not necessarily fully benefited from classroom teaching. However, to certain extent teaching and learning may be influenced by non-linear pedagogy, which is based on manipulating the relevant determining factors (task, environment and individual) to increase information sources and thus guides students towards obtaining their objectives. Within non-linear pedagogy, verbal instruction (e.g., questioning) is considered to be a determining factor that attempts to channel the search for tactical solutions within a learning environment. In order to retain the TGfU, especially the lesser-known revised model and partnership constraints-led approach, in preference to linear traditional pedagogy such as SDT, much greater awareness and understanding of the revised TGfU model and partnership with constraints-led approach among teachers as a practitioners need to be addressed.

Teachers and/or practitioners need to adopt modern evidenced based practice. Through adopting, and then adapting the TGfU revised model and partnership constraints-led approach in doubles badminton game play, players are able to improve their performance especially movement to the base. Notwithstanding, teachers really do need to be aware of, and have an understanding of what is required in order to teach the game of badminton, especially when providing the players with a task that takes environmental constraints into account. Such factors include the interaction between doubles players as a team, opponents, themselves and their opponents strengths and weaknesses, situational setting, community background and so on, which can influence the performer (players). Furthermore teachers must also be aware of the performer (players) as an individual in terms of each individual’s abilities attributes such as speed, agility and shot placement skills among other important components of badminton game play. We, suggest the TGfU revised model and partnership with constraints-led approach needs to be implemented by teachers and practitioners, however, they must understand how to navigate this partnership model effectively.

## Appendix 1

See Table 5.

**Table 5:** Summary of teacher’s reflective journals

Reflection components	Teachers A’s reflection on TGfU model	Teachers B’s reflection on SDT model
Game play in lessons	(i) I did discuss about tactics with the students, such as closing downspace when defending and looking for space when attacking by applying shots such as a drop shot and smash. But at times it was difficult to make the student understand, especially discussing tactics, unless I guided them with clues. (ii) Subsequently they were required to apply some of the tactics discussed in game play, for about 5 min. As they were playing, I moved from one court to another court to discuss the cues about, how to execute skills such as clear, drop shot and smash. As provided in the lesson plan provided by the researcher, I moved on the next game play situation whereby I make them produce the proper skill technique for a clearance, drive, drop shot, and smash while playing in game situations. However, they needed more time and attention to improve their technique of doing the skills. (ii) However, in a game situation the players were able to make right decisions about how to finish off a point and the idea of moving the opposing player around the court trying to get the shuttle into an open space to win, by using forceful shots such as a smash or drop shot	(i) As a teacher, I demonstrated how to serve the shuttle with a proper technique and again I showed them how to clear the shuttle, especially the contact point between shuttle and the racket. Badminton skills were practiced in a skill drills situation, whereby emphasis was given to technique perfection. For each lesson varying activities were involved, namely, from individual activities, followed by pair work, eventually with a double game play to towards end of each lesson. This approach emphasized badminton skills of serve, contact, drive, clear, drop, smash and movement returning to the base. (ii) I must admit that, there were times the players enjoy the badminton skill drills, but at certain of the lesson they experienced fatigue, slowed down the tempo of executing the skill drills including the serve, smash or clearance
Cognitive development	(i) Players were able to identify the name of shots that would send the opponent to the baseline of the court, for example the overhead clear or lob. Sending the shuttle to the baseline, gives the attacking player time to get back into base position. (ii) Throughout many game play situations the players were able to think and then put tactics ideas into action. An example when selecting the type of shot; whether to drop or smash. (iii) While playing double, at times the players were able to understand the common attacking tactics and defending their own court, but at times their concentration lapsed when deciding who would hit the shuttle across the net in a long rally	(i) For each of skill components, teaching cues and learning points were given to the players as they executed the skills of serve, contact, drive, clear, drop, smash and movements when returning to the base. Students asked many questions about how to execute the skills, including for example the serve, and shuttle contact.
Emotional learning	As far emotions are concern, the players were able to smile, shake hands, verbalise expressions such as “ha..a..ha” as they were able to win points On the other hand, when the pair are losing, they still looked overjoyed with their game play	Bye and bye more excitement was indicated as they played mini games at end of each lesson. When they practiced skills in isolated situations, the players, by themselves, were more focusedontheir skill development
Questioning	(i) The implementation of TGfU lessons based on comprehension type questions: ‘Where should your contact shuttle with face of the racket?’‘Why should you position at the base of your own court when to anticipate the return shuttle from opponents?’‘What ways would you kill the opponents rally? and so on led to the fact that most of the question were able to be answered by the students with some teacher guidance. Furthermore they were able to relate their answers to, playing badminton at home, as well as watching their hero Lee Chong Wei playing on television	(i) I asked questions about technical skill execution, as I demonstrated how to execute selected skills (ii) As they are practicing their badminton skills, when I detected their technical errors, I stop them and give the necessary feedback so that they improve the execution of their badminton skills

## Abbreviations

TGfU: Teaching Games for Understanding
SDT: Skill Drill Technical
GBAs: game based approach
WH: Wh questions
